# Isolated thrombocytopenia; A report of a rare presentation of childhood systemic lupus erythematosus (SLE) 

**Published:** 2015

**Authors:** Ahmad Tamaddoni, Behnaz Yousefghahari, Afshin Khani, Mohammadreza Esmaeilidooki, Rahim Barari Sawadkouhi, Iraj Mohammadzadeh

**Affiliations:** 1 Non-Communicable Pediatric Diseases Research Center, Babol University of Medical Sciences, Babol, Iran; 2 Department of Rheumatology, Ayatollah Rouhani Hospital, Babol University of Medical Sciences, Babol, Iran.; 3 Student Research Committee, Babol University of Medical Sciences, Babol, Iran.

**Keywords:** Systemic Lupus Erythematosus, Thrombocytopenia, Pediatrics

## Abstract

**Background:**

Systemic lupus erythematosus (SLE) has various presentations in children. Hematologic abnormalities is common in childhood onset of SLE, however, isolated thrombocytopenia is relatively rare. Thus, we present a child with isolated thrombocytopenia as a rare presentation of SLE.

**Case presentation:**

A 12-year-old boy with chief complaints of loss of appetite, weight loss, decreased platelet count (8000/µL) and lymph node enlargement was referred to our hospital. Biopsy of lymph node showed reactive lymphadenopathy. Investigations regarding infectious disease was negative. Platelet count remained low after low dose steroidned therapy. Antinuclear antibody (ANA) and anti-double stranded DNA antibody screening tests were positive with titer of 1/62 and 1/54, respectively. Therefore, juvenile SLE was considered as the final diagnosis and raising the dose of prednisolone to 2mg/kg/day was associated with increasing platelet count to 40000/µL and a week later to 96000/µL.

**Conclusion:**

The findings of this study indicate that in cases with isolated thrombocytopenia refractory to conventional dose of steroids, SLE should be considered. This study justifies serum ANA and anti DNA assessment in children with thrombocytopenia

**T**he childhood onset of systemic lupus erythematosus (SLE) is relatively rare, and only 20 percent of new cases of SLE are patients under 16 years of age (1). Similar to the adult patients, the prevalence of SLE in children is higher among females, however, due to minimal effect of sex hormones, the female to male ratio is 3:1 which is lower compared to older individuals (2). SLE presentations are diverse in children, however, gradual onset of fever, malaise, small joint arthritis are the most common initial presentations. Hematologic abnormalities are also present in children with SLE including thrombocytopenia, hemolytic anemia, and persistent leukopenia (3). However, these findings are usually accompanied by other well-known signs and symptoms of SLE. However, patients with isolated hematologic abnormalities especially thrombocytopenia as the only presentation of SLE is a rare condition. Since, there are few specific signs and symptoms for the diagnosis of SLE in children and delayed diagnosis is associated with serious complications and poor outcome, early diagnosis and management of the disease is of utmost importance.

Here, we present a boy with isolated thrombocytopenia as the initial presentation of childhood onset SLE.

## Case Presentation

A 12-year-old boy with chief complaints of loss of appetite, weight loss, decreased platelet count (8000/µL) and lymph node enlargement was referred to our institute. His medical history was remarkable for seizure and G6PD deficiency. He was on carbamazepine and phenobarbital for seizure control. His physical examination was unremarkable. Lymph node biopsy was done which revealed reactive lymphadenopathy. 

To rule out infection with pathogens such as mycobacterium tuberculosis, brucella, salmonella, epstein bar virus, h*uman immunodeficiency virus* and leptospira, a thorough laboratory work up was done. His Wright, Widal, p*urified protein derivative *(*PPD*) tests and sputum culture for TB were negative. His *lactate dehydrogenase (LDH) was 667 U/L (normal range=230-460 U/L). *

*His serum complement (C3=91, C4=18 and CH50=76 mg/dl) and total immunoglobulin (IgG=915, IgA=117, IgM=169 and IgE=3 mg/dl) were within normal range. Also, he had a normal* prothrombin and *partial thromboplastin* time**. **Due to inconclusive findings of laboratory work up, bone marrow aspiration was done that had unremarkable findings.

Based on the patient’s data, idiopathic thrombocytopenia (ITP) was considered as the most probable disease and intra venous immune globulin (IVIG) 1 gr/kg/day for 2 days was administered. However, no clinical response was observed. Due to sustained thrombocytopenia, prednisolone 1 mg/kg/day was prescribed which caused no obvious improvement. 

After a week, the patient was admitted again with complaint of hemorrhage. Anti-nuclear antibody (ANA) and anti-double strand DNA antibody screening tests were done and ANA titer was positive 1/62 and anti-double strand DNA antibody titer was positive 1/54.

Therefore, juvenile SLE was considered as the final diagnosis and increasing the dose of prednisolone up to 2mg/kg/day was prescribed to the patient. Platelet count increased up to 40000/µL and a week later to 96000/µL. The patient was referred to rheumatologist for further assessment. Further investigations confirmed the diagnosis as SLE without major organ involvements (table 1). 

**Table 1:** Results of the complementary investigations of the patient

**Para clinic data**	**Result**
**Hematology** White Blood Cell Red Blood Cell Hemoglobin Platelet	18×10^3^ 4.8×10^6^ 13.2 88×10^6^
**Immunology** Lupus Anti-coagulant Anti Cardiolipin IgG Anti Cardiolipin IgM	31 (Negative: 30-45) 3.7 (Negative < 10) 5.6 (Negative < 7)
**Anti-Nuclear Antibody profile** nRNP SM SSA-RO RO-52 SSB-LA SCL-70 JO-1 CENPB DSDNA Nucleosomes Histones Rib. P-protein	Negative Negative Negative Negative Negative Negative Negative Negative Negative Weakley Positive Negative Negative
Urine 24 hours Analysis Protein Creatinine Volume	90 (normal <150 mg/l) 0.7 ( g/day) 2400 ml

## Discussion

The diagnosis of SLE in children is a challenging issue for clinicians so far. Heterogeneous manifestations of SLE and the impact of the disease on the child’s growth highlighted the importance of timely diagnosis and management of SLE in children. Lymphadenopathy was the only common manifestation of SLE in our patient which was reactive in nature. Hematologic abnormalities were reported by other investigators in children with SLE, however, almost always these abnormalities were accompanied by other well-known signs and symptoms of SLE (4, 5). Therefore, the isolated hematologic abnormality in childhood SLE is a relatively rare phenomenon. Campos et al. reported two cases of juvenile SLE that presented with thrombotic thrombocytopenic purpura (TTP). Both of the patients had neurologic, urinary and other specific manifestations of SLE (6). Another case of childhood SLE was reported by Sankar Raj. The patient had fever, abnormal choreiform movements, headache, weight loss and fatigue along with thrombocytopenia (7).

Considering the various presentations of SLE in children, it is essential to diagnose and initiate the appropriate treatment in children to avoid adverse effects of the disease on the patient’s growth. Our report showed that isolated thrombocytopenia which was refractory to conventional dose of steroids may be an early symptom of SLE. Therefore, careful assessment of children with isolated idiopathic thrombocytopenia regarding ANA and dsDNA tests seems to be logical for recognizing SLE as the underlying disease.
